# A sustainable approach to extracting baobab oil: neat supercritical CO_2_ optimization†

**DOI:** 10.1039/d5ra02490k

**Published:** 2025-06-25

**Authors:** Fatlinda Gashi, Charlotta Turner, Arwa Mustafa, Fiona Nermark

**Affiliations:** a Lund University, Department of Chemistry, Centre for Analysis and Synthesis P. O. Box 124 Lund SE-22100 Sweden fiona.nermark@chem.lu.se arwa.mustafa@chem.lu.se

## Abstract

Baobab (*Adansonia digitata*) seeds are a source of valuable lipids with notable nutritional and functional attributes. In response to the rising demand for sustainable, high-quality oils in the cosmetic and nutraceutical sectors, there is an increasing interest in environmentally friendly extraction techniques that maintain lipid bioactivity while reducing the use of toxic solvents. This study represents the first systematic optimization of baobab seed oil extraction utilizing neat supercritical CO_2_ without co-solvents, employing response surface methodology. Under the optimized conditions of 77 °C and a CO_2_ density of 0.8 g mL^−1^, the extracted oil yield was 9.3 ± 1.1 wt%. Although this yield was lower than that achieved through conventional hot pressing (37 wt%), the extracted oil exhibited a fatty acid profile comparable to that of warm-pressed oil, with substantial levels of oleic (37 wt%), linoleic (29 wt%), and palmitic (30 wt%) acids, suggesting higher selectivity for free fatty acids. Furthermore, the scCO_2_ extracted oil retained a solvent-free purity, indicating its suitability for cosmetic and nutraceutical applications. Kinetic studies indicated that solubility, rather than mass transfer, was the primary limiting factor, with an optimal extraction flow rate of 4 mL min^−1^ over 25 minutes. These findings underscore the feasibility and selectivity of neat scCO_2_ extraction as an environmentally friendly alternative to traditional mechanical and solvent-based methods for obtaining high-quality baobab seed oil.

## Abstract 2

Baobab (*Adansonia digitata*) seeds are a source of valuable lipids with notable nutritional and functional attributes.

## Graphical abstract

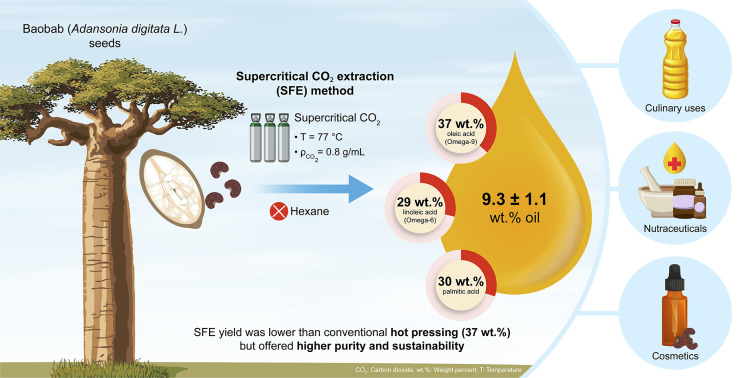

## Introduction

The African baobab, scientifically known as *Adansonia digitata* L., predominantly grows in the sub-Saharan regions of Africa. The baobab is well-known for its highly nutritious fruits, which exhibit a higher content of minerals, fibres, and vitamin C compared to most other fruits.^1^ In 2008, the baobab pulp powder was recognised as a novel food by the European Commission.^2^ The baobab fruit is composed of three primary components: the outer shell (45%), seeds (40%), and fruit pulp (15%).^3,4^ Baobab fruit pulp is valued for its high vitamin C, antioxidants, and dietary fibre content, making it a popular ingredient in juices and as a food additive.^1,5,6^ The leaves of *Adansonia digitata* are rich in proteins, vitamins, and essential minerals such as calcium and potassium.^7^ The leaves are eaten as leafy vegetables in parts of Africa, reducing nutritional deficiencies.^8^ Furthermore, the leaves are employed in traditional medicine due to their antioxidant and anti-inflammatory properties. Baobab seeds are classified as hard seeds because of their tough endocarp, which impedes water and oxygen permeability, contributing to their integumentary dormancy.^9–11^ Baobab seed oil is highly valued in the cosmetic and nutraceutical industries. The oil is rich in essential fatty acids, primarily oleic (omega-9) and linoleic (omega-6) acids, which impart moisturising and skin-nourishing benefits.^12^ It is also abundant in tocopherols, phytosterols, and antioxidants, contributing to its stability, anti-inflammatory, and anti-aging properties.^13^

Despite the growing popularity of baobab products, standardised and environmentally sustainable extraction methods for baobab seed oil remain scarce. Traditional solvent extraction techniques, such as Soxhlet extraction with hexane, offer high efficiency and scalability, but raise significant environmental and health concerns due to the toxicity and flammability of hexane.^14–16^ While cold-press extraction eliminates the use of organic solvents, it frequently results in significantly lower oil yields compared to Soxhlet extraction using hexane. The lower oil yield achieved with cold-press extraction is attributed to incomplete cell disruption and restricted mass transfer.^15^

Supercritical fluid extraction (SFE) has emerged as a promising technique for extracting seed oils, offering environmental advantages and selectivity, while also enhancing oil yield without the use of hexane.^17,18^ SFE with supercritical CO_2_ (scCO_2_) operates at moderate temperatures of 40–80 °C for most applications, making it particularly suitable for preserving thermally sensitive compounds and for producing solvent-free oil.^17,19^ By varying the temperature and pressure, the tunable density of scCO_2_ enables selective extraction of specific compounds, making it a versatile solvent for oil extraction.^20^ Although supercritical carbon dioxide (scCO_2_) extraction has been widely applied to various oil-bearing seeds such as pomegranate,^21^ grape seeds,^19^ and *Moringa oleifera*,^22^ its use for baobab (*Adansonia digitata*) seed oil remains largely unexplored. Existing studies on baobab seed oil have primarily focused on mechanical pressing or organic solvent-based methods such as Soxhlet extraction using hexane.^14–16^ These techniques often pose environmental and health concerns, particularly with the use of toxic solvents. Moreover, the European Food Safety Authority (EFSA) has recently called for a re-evaluation of the use of technical hexane as an extraction solvent due to concerns about potential toxicological risks, including the presence of impurities that may transfer into food and other products.^23^ Therefore, this study was designed as a focused investigation on baobab (*Adansonia digitata*) seeds, which are an underutilised by-product of fruit processing. Conducted in collaboration with Arwa Technologies, the project aimed to develop for the first time, a solvent-free approach to extracting oil from the baobab seed which are a side stream of the company's food production line using neat scCO_2_. In addition to developing and optimising the extraction process through response surface methodology, we also investigated the extraction kinetics and compared the fatty acid profiles of scCO_2_ and warm-pressed oils. This represents the first systematic evaluation of baobab seed oil extraction using supercritical fluid technology, offering new insights into process efficiency and oil quality. To investigate the influence of temperature and CO_2_ density on extractability of oil, we employed Response Surface Methodology (RSM), a statistical tool for modelling multivariable systems.^24^ RSM evaluates individual and interactive effects while minimizing experimental runs. It determined the best conditions for extraction and illustrated the process dynamics. With the help of the optimizer function in the tool, optimum parameters are predicted. Gas chromatography with flame ionization detection (GC-FID) was used to determine the fatty acid composition of the oils, providing crucial information on their nutritional and functional properties. This method allowed for the quantification of major fatty acids like oleic, linoleic, and palmitic acids, which are important indicators of oil quality and potential applications.

## Experimental

### Materials

Ultrapure carbon dioxide (≥99.999%) for supercritical fluid extraction was provided by Linde (Dublin, Ireland). *n*-heptane (≥99%, HiPerSolv chromanorm, for HPLC) and acetonitrile (≥99%, HiPerSolv chromanorm, for HPLC) purchased from VWR chemicals (Leuven, Belgium). Methanolic HCl (3 M in methanol, GC derivatisation grade, LiChropur™ quality) and formic acid ≥ 95% were purchased from Sigma-Aldrich (St. Louis, MO). Supelco 37 component FAME mixture (10 mg mL^−1^ in methylene chloride) was purchased from Supelco (Bellefonte, PA) and ethanol (99.7%) was purchased from Solveco (Rosersberg, Sweden). Water was purified using a Milli-Q purification system (Millipore, Billerica, Ma).

### Sample preparation – sample

The seeds used in the study were supplied by the local company ARWA Foodtech AB (Lund, Sweden). The seeds are a side stream of the company's food production line. Baobab fruits were imported from western Sudan. The pulp-free seeds obtained from the company were dried for 24 hours at 50 °C in a Termark oven. The dried seeds were crushed to a powder with a particle size of >0.17 mm with a Mill MM 200 (Retsch GmbH, Haan, Germany) at 14 000 rpm. The powdered samples were kept in sealed containers in a −20 °C freezer until analysis. Oil from the prepared sample was extracted using scCO_2_ extraction, ultrasonic extraction, and the warm-press method.

### Supercritical CO_2_ extraction

Baobab seed oil was extracted using an analytical SFE system (Waters MV-10, Milford, MA, USA) controlled by ChromScope™ software (Waters, Milford, MA, USA). The system comprised of a fluid delivery module for CO_2_ and co-solvent pumps, an oven, an automated back pressure regulator, a make-up pump, and a fraction collector module. The head of the CO_2_ pump was maintained at 4 °C. About 2 g of seeds powder was loaded into a 5 mL stainless steel extraction vessel fitted with 0.4 mm stainless steel filters at the inlet and outlet. The extraction experiments were all performed in dynamic mode with a constant flowrate of 2 mL min^−1^. At the same time, a make-up flow of *n*-heptane at a rate of 0.5 mL min^−1^ was applied to prevent the precipitation of oil in the lines once the system depressurised and the supercritical CO_2_ turned into gas. Glass tubes were used to collect the extract and the make-up solvent after extraction. The solvent was then evaporated under gentle nitrogen flow at room temperature. The extracts were recorded gravimetrically using weight% and stored in a freezer at −20 °C. Following each extraction, the system was flushed for 30 minutes with a 1 : 1 ratio of ethanol/CO_2,_ followed by a rinse with only CO_2_ for another 15 minutes.

### Method optimization using design of experiment (DoE)

Face-centred central composite designs (FC-CCD) with three centre points was created in MODDE 10.1 (Sartorius Stedim Biotech, Malmö, Sweden) was used to examine the effect of extraction temperature (40–70 °C) and density (0.6–0.8 g mL^−1^) on the extracted gravimetric yield of the oil. An online software, https://webbook.nist.gov/chemistry/fluid/ was used to calculate the density of CO_2_ at different temperatures and pressures used in this study. In total, 11 experiments were performed in a randomised run order (Table S1, ESI†). The model fitting and surface response plot were calculated using multiple linear regression (MLR). The adequacy of the model was evaluated by the *R*^2^ value, which shows the model fit, and *Q*^2^ values, which shows an estimate of the future prediction precision. The predicted *versus* observed plot was used to visualise the difference between the predicted and experimentally measured values. Coefficient and contour plots were used to visualise the effects of temperature and density (variables) on the amount of extracted oil (response) presented as weight percent (wt%). The optimum conditions for extracting the maximum possible amount of oil were determined through numerical and graphical analyses. The criteria of the desirability function set to maximise the amount of oil and the response surface plots were used.

### Reference extraction methods

#### Ultrasonic extraction

Ultrasonic extraction was conducted in batch mode following a method described by Belo *et al.*, with some modifications.^25^ The extraction was performed by mixing 2 g of seed powder with 25 mL *n*-heptane in a 100 mL conical flask, which was then treated in a sonication water bath for 20 min at 60 °C. The extract was then transferred to a 50 mL falcon tube and centrifuged at 20 °C for 10 minutes at 2500 rpm. The supernatant was collected, the residue was subjected to new extraction with fresh solvent and the same extraction procedure was repeated twice. The extracts were pooled, and the solvent was evaporated under a gentle nitrogen flow at room temperature. The extraction was performed in triplicate. The oil yield was calculated by dividing the mass of oil extracted by the initial mass of dried baobab seeds and multiplying it by 100 to express the result as a percentage.

#### Warm press

The seeds were processed at Gunnarshögs Gård AB, Österlen, Sweden, utilising standard warm-press technology for rapeseeds, with temperature adjustments.^26^ The hydraulic pressing apparatus was heated to 90 °C. A batch of 400 g baobab seeds was subjected to extraction. The press was operated continuously until all seeds were extracted. The extracted oil samples were collected in containers and stored at 4 °C in a refrigerator until further analysis. The extraction was performed in triplicates. The oil yield was calculated by dividing the mass of oil extracted by the initial mass of dried baobab seeds and multiplying by 100 to express the result as a percentage.

#### FAMEs analysis by GC/FID

The extracts obtained using the optimised parameters from SFE were analysed on a GC-FID (model 7890 B, Agilent Technologies, Inc., Wilmington, DE, USA) equipped with a capillary HP-5 column (30 m × 320 μm inner diameter (i.d.) × 0.25 μm film thickness) from Agilent Technologies. The system was controlled using Agilent MSD ChemStation software. The chromatographic separation was adopted from a published method by Mező *et al.*, with some modifications.^27^ The separation was as follows: initial ramp from 50 °C to 170 at 20 °C min^−1^ and held for 4 min, then increased to 190 °C at a rate of 3 °C min^−1^ and held for 5 min, then increased to 240 °C at a rate of 3 °C min^−1^, held for 3 min, then increased to 290 °C at a rate of 8 °C min^−1^ and held for 2 min, then increased to 310 °C at a rate of 10 °C min^−1^ and held for 2 min. Finally, the temperature was increased to 320 °C at the rate of 40 °C min^−1^. The GC injector port temperature was maintained at 250 °C, and the spitless injection mode was used throughout the analysis. The air flow rate in the FID detector was set to 400 mL min^−1^, hydrogen flow rate was set to 30 mL min^−1^, and make-up gas flow rate was set to 25 mL min^−1^. The extracts were reconstituted with 1 mL *n*-heptane, and the injected volume was 1 μL. Fatty acid methyl esters (FAMEs) in the baobab seed oil were identified by retention times compared to an external standard (Supelco® 37 Component FAMEs Mix). Additionally, to further confirm the identification, linear retention indices (RIs) of the FAMEs were also calculated using a mixture of hydrocarbons (*n*-dodecane to *n*-hexatriacontane). The concentration of each fatty acid was determined by integrating chromatographic peak areas. Peak areas were used to calculate FAMEs concentrations in the oil, expressed in micrograms per millilitre (μg mL^−1^) based on the standard mix calibration curve. To express results as percentage composition, the measured concentration (μg mL^−1^) of each FAME was converted to mass fraction by normalising to total oil analysed. The relative abundance was calculated as weight percentage (wt%) of total oil. This method enables comparison of fatty acid profiles across extraction methods and interpretation of extraction selectivity.

## Results and discussion

Baobab (*Adansonia digitata*) seeds, a by-product of food production, are abundant in valuable lipids.^28^ As outlined in the introduction, Soxhlet extraction utilizing hexane is a prevalent method for oil recovery from baobab seeds. However, the use of hexane presents a significant drawback due to its toxicity and the potential for residual presence in the extracts. To our knowledge, this study is the first to report the use of neat supercritical carbon dioxide (scCO_2_) for the extraction of baobab seed oil without the inclusion of co-solvent modifiers. This method is distinct from previous supercritical fluid extraction (SFE) studies that incorporate ethanol or other solvents to enhance oil recovery. The current approach ensures the production of solvent-free oil with high compositional quality and environmental compatibility.

### Optimisation of the extraction parameters – extracted amount

The impact of density and temperature on seed oil yield in the extraction process was investigated using face-centred central composite design (FC-CCD). In the design of experiments (DoE), density was chosen as an independent variable instead of pressure because density directly influences the extraction efficiency by modulating the solvent polarity of supercritical carbon dioxide (scCO_2_). Pressure alone does not directly affect the extraction efficiency; it alters the density. When temperature and pressure are used as independent variables, the resulting density values became nonlinearly distributed across the experimental points, complicating the interpretation of the extraction process. The variables under investigation, their ranges, and the responses obtained in each experiment are listed in Table S1 (ESI†). The investigated ranges were selected based on the extraction instrument's limitations. The amount of dried sample (2.0 g), extraction flow rate (3.0 mL min^−1^), and make-up solvent flow rate (0.5 mL min^−1^) were kept constant.

The results showed that extracting oil from baobab seeds using neat scCO_2_ as the extraction solvent provided a valid model (Fig. S1, ESI†) with *R*^2^ = 0.98, which describes the model fit and *Q*^2^ = 0.92, which estimates the future prediction precision. Any model with a *Q*^2^ value of more than 0.25 is considered valid and has good prediction power. A comparison of the predicted and observed values (Fig. S2, ESI†) demonstrated the model's reliable predictive ability, as the data points were clustered around the diagonal line.

**Fig. 1:**
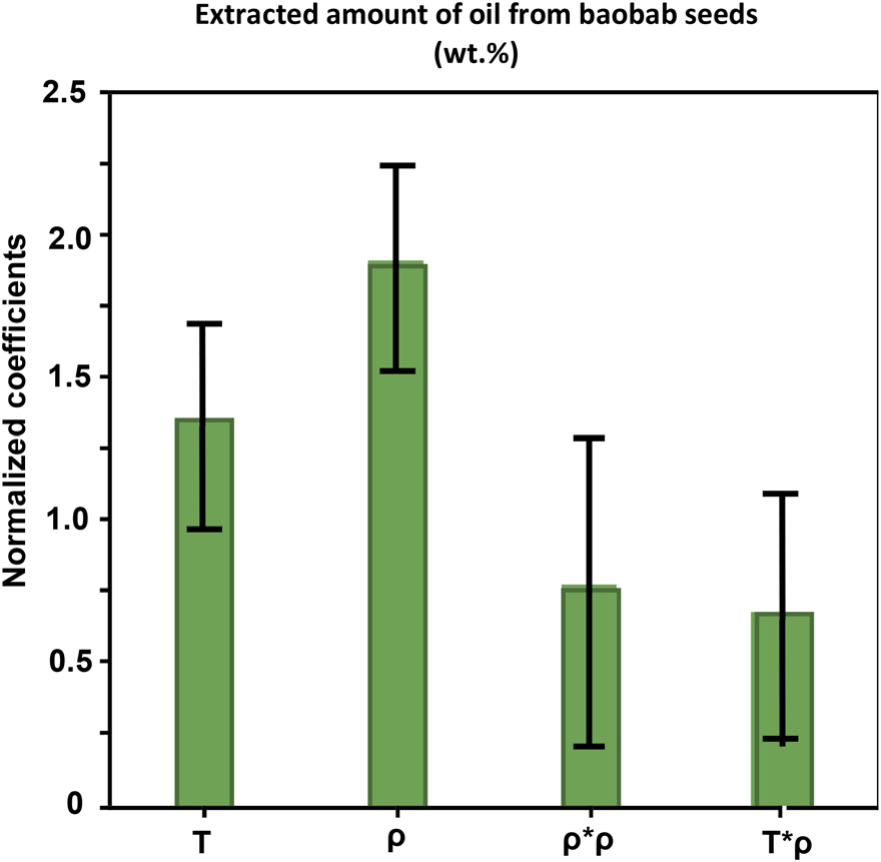
Coefficient plot showing the influence of temperature (*T* °C) and density (g mL^−1^) as well as two-factor interactions on the extracted amount (weight%) of the baobab seed oil using neat scCO_2_.

A coefficient plot (Fig. 1) was used to visualise interactions between variables. The coefficient plot shows that temperature (*T*) and density (*ρ*) have a strong positive effect (positive coefficient) on the extraction of the total oil. Furthermore, the strong interaction between *ρ* × *ρ* and *T* × *ρ* with positive coefficients indicates that increasing the temperature and density increases the amount of extracted oil.

**Fig. 2:**
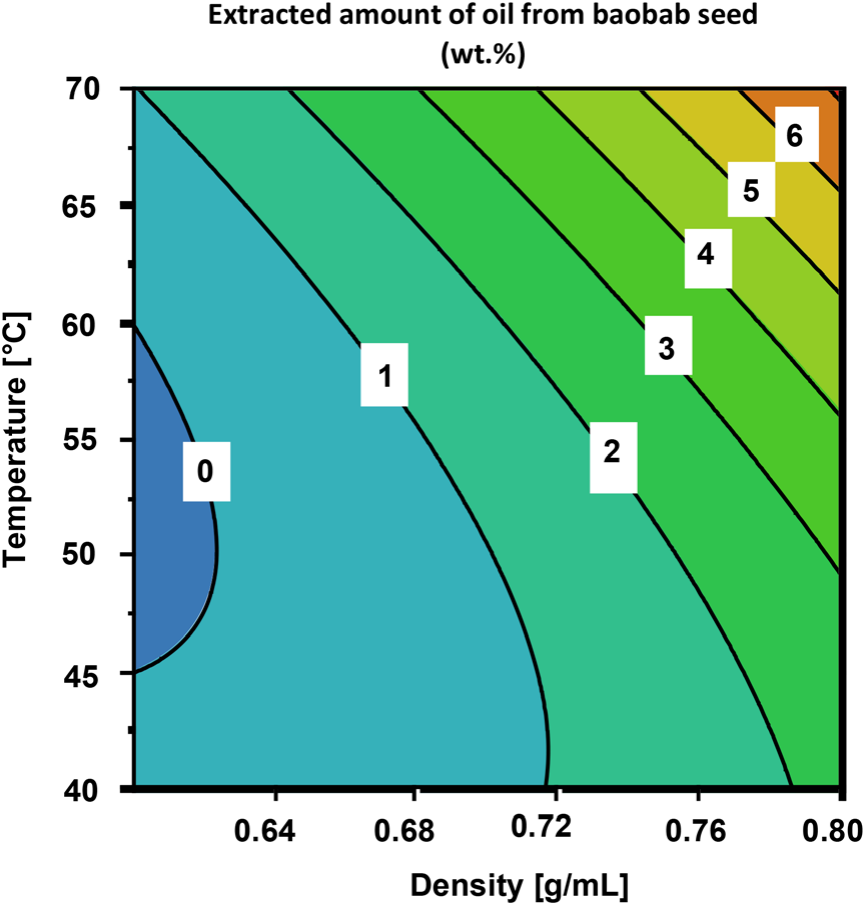
Contour plot showing the extracted amount of baobab oil in weight% at varied temperatures and densities of scCO_2_.

The total amount of the extracted oil varied from 0.25 to 6.4 wt%, with different extraction conditions (Table S1, ESI†). The trend observed in the contour plot in Fig. 2 shows increasing the density of scCO_2_ from 0.6 to 0.8 g mL^−1^ and the temperature from 40 to 70 °C generally resulted in extracted amount of oil. The extractability of oil from seeds was enhanced by increasing the density of scCO_2_. This is attributed to the reduced intermolecular distance between CO_2_ molecules, resulting in a higher overall electron density and increased solvation.^25,29^ Moreover, increased pressure may lead to greater rupture of cell walls, thereby facilitating solvent penetration into the seed matrix, which enhances oil extraction.^30^ Similar results were observed by Tanveer *et al.*, who investigated the effect of pressure on the extraction yield of fennel essential oils from Egypt and Pakistan. They reported an increase in the amount of oil from 2 wt% to 3 wt% and 4 wt% when the pressure was increased from 172 bar to 241 bar at 60 °C.^31^ Belo *et al.* reported similar findings in which the extracted amount of moringa seed oil increased from 285 mg to about 395 mg when pressure was increased from 400 to 800 bar at a constant temperature of 55 °C, which was explained by the increment in the density of the scCO_2_ (from 0.91 to 1.03 g mL^−1^).^25^ Their results also showed that increasing temperature also resulted in increased amount of total oils extracted, which might be due to the increase in the vapor pressure of the oils.^32^

The optimum conditions required to achieve the highest amount of oil were estimated using an algorithm based on a desirability function in the design optimiser in the MODDE® software.^33^ The optimum conditions were predicted to be a temperature of 70 °C at an scCO_2_ density of 0.8 g mL^−1^. The optimum temperature was found at the corner of the design space where both the temperature and density were the highest. This observation suggests that the actual optimum temperature and density required to extract the maximum amount of oil from baobab seeds are outside the design space of this study. An additional experimental point was performed in triplicate, where the density was maintained at 0.8 g mL^−1^ and the temperature was increased to 77 °C to investigate whether the oil yield would increase. The results are presented in Table 1. The temperature was chosen because the pressure could be maintained at 350 bar and the maximum SFE system could withstand. The amount of extracted oil increased from 6.4 wt% (highest yield obtained with DoE) to 9.3 ± 1.1 wt%, as shown in Table 1. An increase in temperature leads to an elevation in vapor pressure, thereby enhancing the solubility of oils within the seeds.^34^

**Table 1:** Comparison of the new neat scCO_2_ extraction method and selected conventional methods from the literature for the extraction of oil from baobab seeds. For neat CO_2_, ultrasonic extraction method, and warm press, the extracted amount was calculated as % dry extract weight/initial sample weight, presented as weight% (wt%)

Extraction method	Extraction solvent	Temperature (°C)	Pressure (bar)	Extraction time (minutes)	Organic solvent usage (mL)	Extracted amount (wt%)	Reference
Supercritical fluid extraction	Neat CO_2_	77	350 bar	20	0	9.3 ± 1.1 (*n* = 3)	This study
Ultrasonic extraction	*n*-Heptane	60	Atmospheric pressure	60	75	12.4 ± 0.7 (*n* = 3)	This study, adopted from Belo *et al.*, 2019 (ref. 25)
Soxhlet	*n*-Hexane	60	Atmospheric pressure	360	600	27.8	Ofori *et al.*, 2023 (ref. 15)
Cold press	No solvent	Room-temperature	Atmospheric pressure	Not specified	0	5.4	Ofori *et al.*, 2023 (ref. 15)
Warm press	No-solvent	90	Atmospheric pressure		0	37 ± 3 (*n* = 3)	This study

### scCO_2_ extraction kinetics

**Fig. 3:**
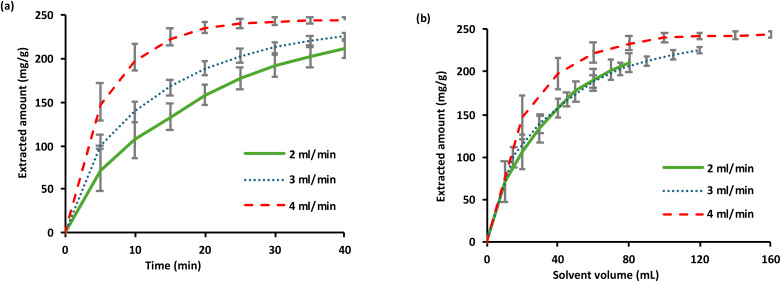
Extracted amount of baobab seed oil (mg g^−1^ of baobab seed powder) *versus* extraction time (a) and solvent volume (b) at three different flow rates. The extraction temperature was 77 °C, and the density of scCO_2_ was 0.8 g mL^−1^.

Studying the extraction kinetics is essential for optimising the scCO_2_ extraction process by identifying the rate-limiting step and determining the most efficient operational conditions. In this study, the flow rate was assessed at three distinct levels: 2, 3, and 4 mL min^−1^, under conditions that resulted in maximum oil extraction (77 °C and 0.8 g mL^−1^). Factions were collected at 5 min intervals for 40 min. The extraction kinetics curves are shown in Fig. 3. These curves illustrate the cumulative yield as a function of the extraction time (Fig. 3a), and solvent volume used (Fig. 3b). In the first 10 min, the extracted amount increased linearly with flow rate (Fig. 3a). Moreover, the curves exhibited a significant overlap during the initial phase of the extraction process when the extracted quantities were plotted against the solvent volumes (Fig. 3b). Consequently, solubility serves as the limiting factor in the extraction process, rather than mass transfer. This observation indicates that the oil rapidly moved through the sample matrix, but its solubility hindered its ability to dissolve the oil. Therefore, it can be assumed that the amount of extracted oil per unit time would increase if the extraction was performed at a higher flow rate. To reduce the extraction time, it is suggested that the extraction be performed at the maximum flow rate during the initial extraction period (0–15 min). The extracted amount per unit time decreased after 15 min, suggesting that mass transfer of the compounds within the sample matrix limits the extraction rate. Because the partitioning of the analytes depends on the distribution constant, this might also suggest that the concentration difference between the sample and solvent is negligible, as there might only be trace amounts of extractable compounds remaining in the sample. Overall, Fig. 3 shows that the extraction rate is controlled by the solubility of oil in the scCO_2_ solvent. As a result, the ideal flow rate and extraction time for baobab seed oil extraction were estimated to be 4 mL min^−1^ and 25 min, respectively.

### Comparison with other extraction methods – extracted amount

The efficiency of the proposed supercritical fluid extraction (SFE) method using neat scCO_2_ at 77 °C and 350 bar was evaluated by comparing it with two previously reported methods. Ultrasound-assisted extraction (UAE) with heptane as the solvent was used to extract oil from *Moringa oleifera* seeds by Belo *et al.*,^25^ The warm press methods has been used to extract oil from rapeseeds.^26^ In addition, extracted amounts using Soxhlet extraction with hexane as the solvent and cold-press yield reported by Ofori *et al.* are also compared to the results from this study.^15^ A summary of these methods and their corresponding yields is provided in Table 1. The extracted amount from extraction with scCO_2_ using the best conditions was 9.3 ± 1.1 wt%, lower than the reference methods based on warm press (37 ± 3 wt%) and ultrasonic extraction 37 ± 3 wt%. One of the properties of supercritical CO_2_ is its selectivity. Consequently, the lower yield may be attributed to the limited solubility of highly polar lipids in supercritical CO_2_ under extraction conditions. UAE with *n*-heptane at 60 °C resulted in a yield of 12.4 ± 0.7 wt%, which, while higher than scCO_2_ extraction, remained lower than Soxhlet extraction reported in literature (Table 1).^15^ Soxhlet extraction typically provides higher yields because it involves continuous solvent cycling at the solvent's boiling point for extended periods, allowing for thorough and exhaustive extraction of oils from the seed matrix. Warm pressing at 90 °C resulted in the highest yield (37 ± 3 wt%), significantly outperforming scCO_2_ extraction and UAE. This result highlights the effect of elevated temperatures on reducing the oil viscosity and facilitating oil release. Cold pressing at room temperature resulted in a significantly lower yield (5.4 to 6.3 wt%),^15,35^ emphasising the importance of heat in increasing oil extraction efficiency.

### Fatty acids composition

Determining the fatty acid composition of baobab seed oil is essential to evaluate its nutritional, functional, and industrial potential. In this study, the extraction efficiencies of baobab seed oil using neat supercritical carbon dioxide (scCO_2_), ultrasonic extraction with heptane, and warm pressing were compared. Additionally, the composition of fatty acid methyl esters (FAMEs) in oils extracted using neat scCO_2_ under optimised conditions (Fig. S3, ESI†), and the warm press method was analysed using gas chromatography with flame ionisation detection (GC/FID) to assess the FAMEs profile. These two methods were compared because they do not employ organic solvents. Moreover, it was also interesting to determine whether the warm press extracted more FAMEs than scCO_2_ because the overall extracted amount was significantly higher that scCO_2_ extracted oil. The fatty acid composition of baobab seed oil extracted using neat scCO_2_ (SFE) and the warm-press method showed minor differences in lipid profiles (see Table 2). Both methods extracted oils with high oleic acid content, measuring 37.0 ± 1.20 wt% for SFE and 37.6 wt% for warm press. Linoleic acid was extracted at 29.0 ± 0.75 wt% using SFE and 28.9 wt% with warm-press, consistent with previously reported compositions of baobab seed oil.^28,36^ A notable difference is the higher extracted amount of palmitic acid by SFE (30.0 ± 0.90 wt%) compared to the warm-press method (23.4 wt%). This difference may be attributed to the selective solubility of supercritical CO_2_, which preferentially extracts specific lipid fractions based on the density and temperature. Increased extraction efficiency of palmitic acid under supercritical conditions has been observed in previous studies on lipid extraction,^25^ suggesting that SFE may enhance the recovery of certain saturated fatty acids. The warm press method resulted in slightly higher amounts of behenic acid (0.4 wt%) than SFE (0.27 ± 0.02 wt%). This could be due to the mechanical pressing process at higher temperature of 90 °C compared to 77 °C used in SFE, which may extract more high-molecular-weight lipids that would require additional energy input for solubilization in scCO_2_.^37^ Similarly, minor fatty acids such as myristic acid (0.29 ± 0.01 wt% in SFE *vs.* 0.2 wt% in warm-press) and *cis*-10-heptadecenoic acid (0.40 ± 0.02 wt% in SFE *vs.* <0.1 wt% in warm-press) were more efficiently extracted using scCO_2_, possibly due to the optimized solubility conditions in the supercritical phase. The differences in fatty acid composition between these two methods align with findings from previous research, where SFE was shown to selectively extract medium-chain and unsaturated fatty acids more efficiently than mechanical pressing.^38,39^ The ability of the scCO_2_ method to operate at low temperatures (77 °C) may minimise thermal degradation and preserve the integrity of polyunsaturated fatty acids such as linoleic acid. On the other hand, the warm-press method may cause slight thermal degradation or oxidation of sensitive fatty acids, potentially altering the final lipid profile.^37^ Apart from the FAMEs profile, the overall total extracted amount of FAMEs by the scCO_2_ and warm press is not very different despite the large difference in the total extracted amount. This observation shows that scCO_2_ is more selective than warm-pressing.

**Table 2:** Fatty acid composition of baobab seed oils extracted by SFE using neat scCO_2_ at optimised conditions and warm press reference method, expressed as weight percent (wt%) of total extracted oil indicated in Table 1 (*n* = 3)

Fatty acid	scCO_2_ extraction (developed method) wt%	Warm press (reference method) wt%
Myristic acid	0.29 ± 0.01	0.2
Pentadecanoic acid	0.06 ± 0.00	<0.1
Palmitoleic acid	0.28 ± 0.01	0.1
Palmitic acid	30.0 ± 0.90	23.4
*cis*-10-Heptadecenoic acid	0.40 ± 0.02	<0.1
Heptadecanoic acid	0.22 ± 0.01	0.2
Linoleic acid	29.0 ± 0.75	28.9
Oleic acid	37.0 ± 1.20	37.6
Stearic acid	4.10 ± 0.20	3.7
Arachidic acid	0.94 ± 0.06	<0.1
Behenic acid	0.27 ± 0.02	0.4
Tricosanoic acid	0.06 ± 0.01	<0.1
Lignoceric acid	0.19 ± 0.02	0.2

## Conclusion

In this study, a solvent-free supercritical CO_2_ extraction method for baobab seed oil was developed and optimised using response surface methodology. The optimal extraction conditions, identified as 77 °C and a CO_2_ density of 0.8 g mL^−1^, resulted in an oil yield of 9.3 ± 1.1 wt%. Although this yield was lower than that obtained using conventional methods such as hot pressing (37 wt%), supercritical fluid extraction (SFE) offered significant advantages in terms of sustainability and extract purity. The fatty acid profile of the SFE extract was comparable to that of hot-pressed oil, exhibiting high levels of oleic acid (37 wt%), linoleic (29 wt%), and palmitic (30 wt%) acids, highlighting its potential applications in the cosmetics and nutraceutical industries. Furthermore, scCO_2_ demonstrated selectivity for specific fatty acids, distinguishing it from warm-pressing. Analysis of the extraction kinetics revealed that solubility, rather than mass transfer, was the primary factor governing oil recovery, highlighting the importance of optimising the temperature and solvent flow rate. Based on the kinetic studies, a flow rate of 4 mL min^−1^ for 25 min was recommended. While further studies could enhance yields, the current findings establish SFE using neat CO_2_ as a viable method for producing high-purity baobab seed oil. This method offers a sustainable, solvent-free alternative to traditional extraction techniques, generating a high-quality oil extract suitable for diverse applications in the cosmetic, nutraceutical, and food industries. Although the technical optimisation and compositional evaluation of baobab seed oil extraction using neat scCO_2_ were the main focus of this study, a thorough economic analysis and cost comparison with traditional methods is advised as a crucial avenue for future research, especially when assessing feasibility on an industrial scale.

## Author contributions

FG performed experiments and contributed to writing the first draft of the manuscript. CT and AM conceptualised and critically discussed the results of the study. FN contributed to experimental work and wrote the first draft of the manuscript with FG. All authors contributed to writing the final version of the manuscript.

## Conflicts of interest

There are no conflicts to declare.

## Supplementary Material

RA-015-D5RA02490K-s001

## Data Availability

The data supporting this article have been included as part of the ESI.†
