# Supplementary material for: A sustainable approach to extracting baobab oil: neat supercritical CO2 optimization
Source: RSC Adv. 2025 Jun 25;15(27):21803–10. doi: 10.1039/d5ra02490k (PMC12189197; doi:10.1039/d5ra02490k)
Supplement: RA-015-D5RA02490K-s001 [file RA-015-D5RA02490K-s001.pdf]

Supplementary Information (SI) for RSC Advances.
This journal is © The Royal Society of Chemistry 2025

A Sustainable Approach to Extracting Baobab Oil: Neat Supercritical CO_2 Optimization

Fatlinda Gashi,[a] Charlotta Turner,[a] Arwa Mustafa *[a] and Fiona Nermark,*[a]

[a] Chemistry, Lund University, P. O. Box 124, Lund, SE-22100, Sweden

* Correspondence: fiona.nermark@chem.lu.se and arwa.mustafa@chem.lu.se

Table S1. Experimental conditions were obtained by using a face-centred central composite design and total oil (yield, % w/w) in the extracts obtained under indicated temperature (°C) and density (g/mL). The pressure (bars) applied to achieve the desired density is also shown.

Exp name	Run order	Temperature (°C)	Density (g/mL)	Pressure (bar)	Extracted amount (%, w/w)
N1	6	40	0.6	97	0,3
N2	2	70	0.6	176	1,4
N3	10	40	0.8	164	2,6
N4	9	70	0.8	315	6,4
N5	11	40	0.7	114	0,6
N6	8	70	0.7	223	3,8
N7	5	55	0.6	136	0,8
N8	3	55	0.8	239	4,9
N9	4	55	0.7	168	1,7
N10	7	55	0.7	168	2,1
N11	1	55	0.7	168	1,5

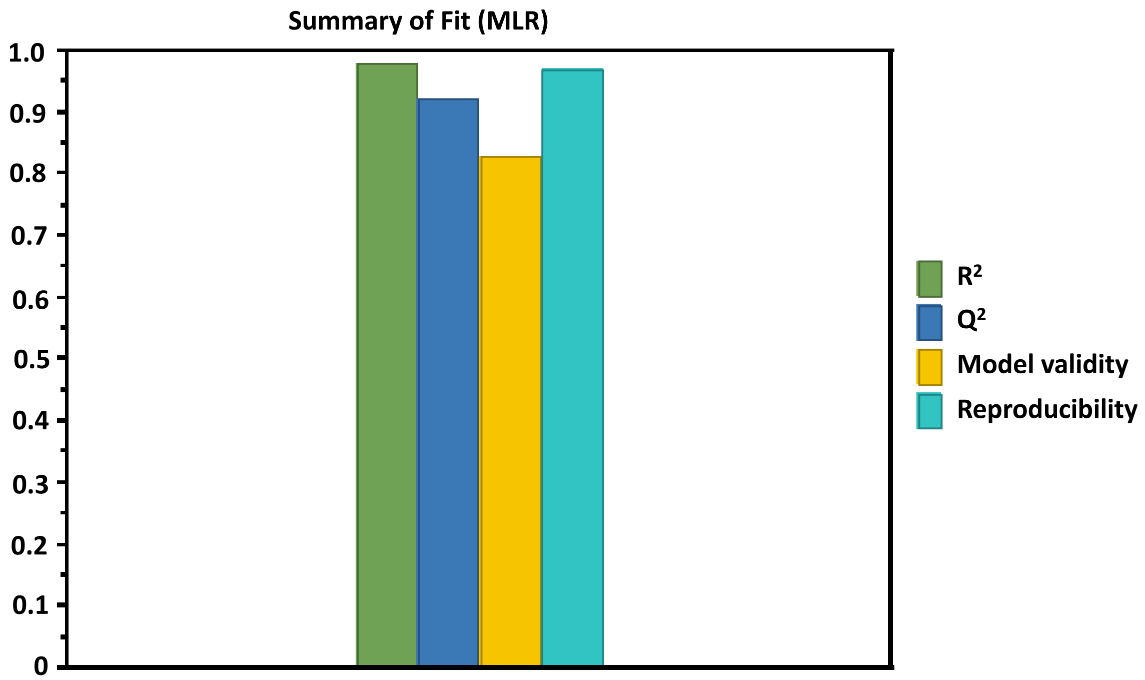

Figure S1. Summary of fit on multiple linear regression. DoE model fitting shows the R^2 (98%) and Q^2 (92%) values with validity and reproducibility evaluations.

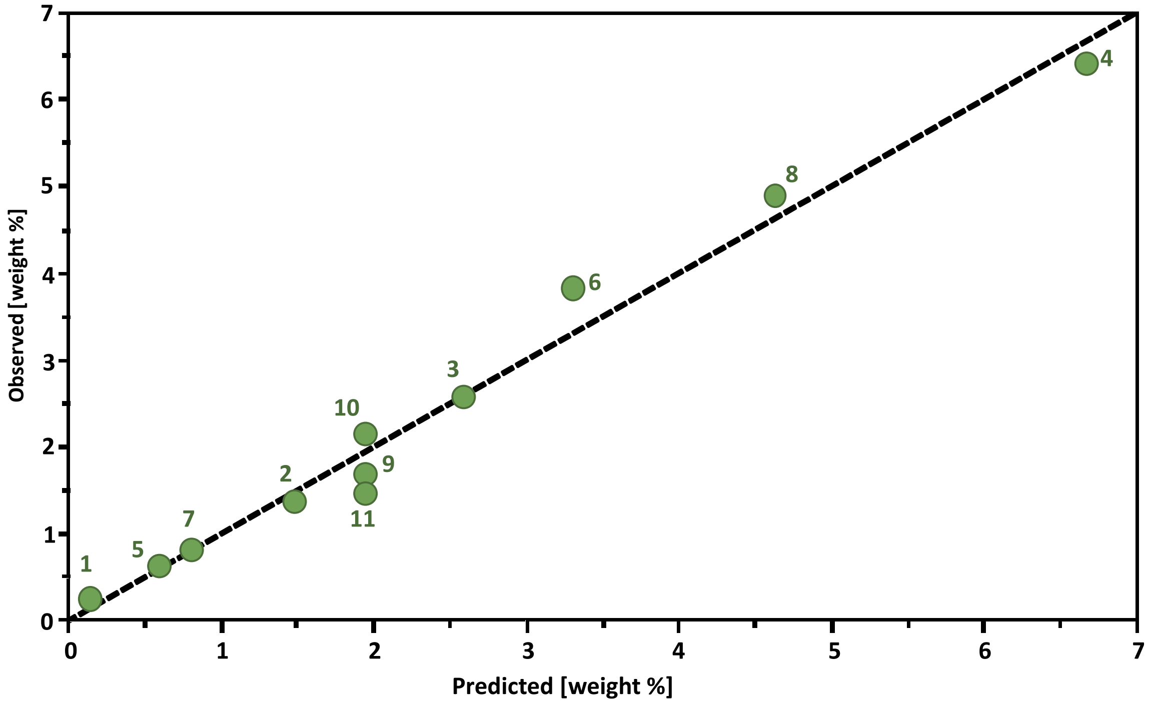

Figure S2 Plot shows the linearity relationship between the observed vs predicted values from the model for extraction of baobab seed oil using neat supercritical CO_2 as a solvent .
